# Optimizing Recellularization of Whole Decellularized Heart Extracellular Matrix

**DOI:** 10.1371/journal.pone.0090406

**Published:** 2014-02-27

**Authors:** Matthew J. Robertson, Jessica L. Dries-Devlin, Stefan M. Kren, Jana S. Burchfield, Doris A. Taylor

**Affiliations:** 1 Center for Cardiovascular Repair, University of Minnesota, Minneapolis, Minnesota, United States of America; 2 Department of Molecular Cardiology, Texas Heart Institute, Houston, Texas, United States of America; 3 Medtronic, Mounds View, Minnesota, United States of America; 4 Department of Regenerative Medicine Research, Texas Heart Institute, Houston, Texas, United States of America; 5 Department of Integrative Biology and Physiology, University of Minnesota, Minneapolis, Minnesota, United States of America; Georgia Regents University, United States of America

## Abstract

**Rationale:**

Perfusion decellularization of cadaveric hearts removes cells and generates a cell-free extracellular matrix scaffold containing acellular vascular conduits, which are theoretically sufficient to perfuse and support tissue-engineered heart constructs. However, after transplantation, these acellular vascular conduits clot, even with anti-coagulation. Here, our objective was to create a less thrombogenic scaffold and improve recellularized-left ventricular contractility by re-lining vascular conduits of a decellularized rat heart with rat aortic endothelial cells (RAECs).

**Methods and Results:**

We used three strategies to recellularize perfusion-decellularized rat heart vasculature with RAECs: retrograde aortic infusion, brachiocephalic artery (BA) infusion, or a combination of inferior vena cava (IVC) plus BA infusion. The re-endothelialized scaffolds were maintained under vascular flow *in vitro* for 7 days, and then cell morphology, location, and viability were examined. Thrombogenicity of the scaffold was assessed *in vitro* and *in vivo*. Both BA and IVC+BA cell delivery resulted in a whole heart distribution of RAECs that proliferated, retained an endothelial phenotype, and expressed endothelial nitric oxide synthase and von Willebrand factor. Infusing RAECs via the combination IVC+BA method increased scaffold cellularity and the number of vessels that were lined with endothelial cells; re-endothelialization by using BA or IVC+BA cell delivery significantly reduced *in vitro* thrombogenicity. *In vivo*, both acellular and re-endothelialized scaffolds recruited non-immune host cells into the organ parenchyma and vasculature. Finally, re-endothelialization before recellularization of the left ventricular wall with neonatal cardiac cells enhanced construct contractility.

**Conclusions:**

This is the first study to re-endothelialize whole decellularized hearts throughout both arterial and venous beds and cavities by using arterial and venous delivery. The combination (IVC+BA) delivery strategy results in enhanced scaffold vessel re-endothelialization compared to single-route strategies. Re-endothelialization reduced scaffold thrombogencity and improved contractility of left ventricular-recellularized constructs. Thus, vessel and cavity re-endothelialization creates superior vascularized scaffolds for use in whole-organ recellularization applications.

## Introduction

Heart disease is the leading cause of death in the United States and comprises a spectrum of disorders from congenital defects to diseases that impair the heart's limited potential to repair itself [1]. Cardiac tissue engineering holds promise for repairing congenital heart defects [2], replacing diseased aortic valves [3], and restoring scarred myocardial tissue [4]. In addition, cardiac tissue engineering can be used to generate tissue “patches” that provide support to the ventricular wall and enable delivery of reparative stem/progenitor cells to damaged myocardium [5]–[8]. Eventually, cardiac tissue engineering may even be used to create a transplantable whole heart from a patient's own stem/progenitor cells.

For cardiac tissue engineering to reach its full clinical potential, engineered tissues and organs must be structurally and functionally similar to healthy myocardium [9]. The myocardium is a dense highly vascular tissue that is sensitive to ischemia and has a thickness of up to one centimeter [10]. Engineered cardiac tissues will have to be highly vascularized like the native myocardium–with nearly one capillary per cell–to support the high rate of cardiomyocyte oxygen consumption and to prevent ischemia within the construct. In addition, the engineered cardiac tissue should integrate into the native circulation or existing heart after transplantation. Relying on diffusion alone to support a thick cardiac tissue–engineered construct is insufficient to compensate for the lack of a vasculature because diffusion cannot support tissues thicker than 100 microns [11]. To overcome a lack of vascularization in engineered constructs, previous approaches have relied on the use of porous synthetic scaffolds [12], the ingrowth of new vessels from the recipient into the construct [13]–[17], or scaffolds that have a pre-existing vasculature [18]–[20].

Acellular scaffolds generated from cadaveric hearts have not only a pre-existing vasculature with a high ratio of vessel conduits to parenchyma, but also a chemical composition, mechanical properties, and a scaffold geometry that are similar to native heart tissue [20]. However, acellular vessel conduits and naked endocardium are thrombogenic and are unlikely to be usable as perfusable tissue constructs without an endothelium. However, endothelial cells must be delivered in a manner that appropriately localizes them to the vascular conduit surfaces and the ventricular cavity, not to the parenchyma of the scaffold.

We and others have shown that perfusion decellularization can be applied to cadaveric rat, mice, and pig hearts to create acellular scaffolds that have patent and perfusable vessel conduits [20]–[24]. Moreover, these scaffolds have been used to generate nascent, beating, drug-responsive heart constructs [20], [24]. Although heterotopic transplantation of these acellular scaffolds is possible, the scaffolds are thrombogenic even with anti-coagulation (data not shown) [20]. In the present study, we build on our previous work to show that perfusion-decellularized acellular scaffolds can be re-endothelialized with functional endothelial cells, which reduces the thrombogenicity of the scaffold. Moreover, re-endothelialization improves contractile function of constructs that have been re-cellularized. These re-endothelialization studies are a first step toward generating an engineered functional arterial and venous vasculature that can be used to create transplantable, viable tissues and organs.

## Methods

### Animals

All experiments were performed in accordance with the US Animal Welfare Act and were approved by the Institutional Animal Care and Use Committee at the University of Minnesota. Heart matrices were derived from female Sprague Dawley rats (9–20 weeks old, Harlan Laboratories) or female Fischer 344 rats (9–16 weeks old, Harlan Laboratories). In the transplantation experiments, male and female athymic Hsd: RH-*Foxn1^rnu^* nude rats (7–13 weeks old) (Harlan Laboratories) received a heart matrix derived from Fischer 344 rats. All rats used in the generation of heart scaffolds were anesthetized with an intraperitoneal injection of 100 mg/kg ketamine and 10 mg/kg xylazine before systemic heparinization and subsequent removal of the heart. In the transplantation experiments, recipient rats were anesthetized with sodium pentobarbital (60 mg/kg).

### Decellularization of cadaveric rat hearts

Cadaveric rat hearts were decellularized by coronary perfusion as previously described [20]. Briefly, rats were anesthetized and heparinized, and a median sternotomy was performed. The pericardium was dissected and retrosternal fat was removed to expose the mediastinal vessels. The first three branches of the ascending thoracic aorta were ligated and transected as were both superior vena cavae. After transecting the inferior vena cava (IVC) and the pulmonary vessels, we removed the heart from the thoracic cavity and placed it in a petri dish containing phosphate-buffered saline (PBS). Then, the heart was catheterized and flushed with PBS. Finally, we gravity perfused the hearts with 1% sodium dodecyl sulfate (SDS) overnight at about 80 mmHg and washed them with deionized water, 1% Triton-X100 (Sigma), and antibiotic-containing PBS (100 U/mL penicillin, 100 U/mL streptomycin; Life Technologies). Immediately after decellularization, scaffolds were transferred to a tissue culture incubator and pre-conditioned using retrograde aortic perfusion of complete MCDB-131 medium (Vec Technologies) overnight at 37°C.

### Re-endothelialization of rat heart scaffolds

Rat aortic endothelial cells (RAECs) (Vec Technologies) were used in all re-endothelialization experiments. RAECs were cultured on gelatin-coated T185 flasks in complete MCDB-131 medium and passaged using TrypLE Express (Life Technologies). To determine the optimal method of re-endothelialization, we used three different strategies to deliver RAECs into the acellular scaffolds: 1) direct aortic infusion of cells, 2) infusion of cells into the brachiocephalic artery (BA), or 3) a combination of venous (via the IVC) and arterial (via the BA) cell infusions. For the aortic infusion, we stopped retrograde aortic media perfusion of the scaffolds, cannulated the aorta distal to the third branch of the aorta, and perfused 2.0×10^7^cells into the decellularized scaffolds. Cells were allowed to attach for 1 hour before constructs were re-cannulated and perfused via the aorta with complete MCDB-131. For BA infusions, we cannulated the BA and perfused either 2.0×10^7^ cells or 4.0×10^7^ cells. During the BA infusions, constructs were kept under retrograde aortic perfusion of complete MCDB-131. For the combination strategy, we stopped retrograde perfusion of media via the aorta and cannulated the IVC. Next, we infused 2.0×10^7^ cells, placed the scaffolds under retrograde perfusion of medium via the aorta, and infused 2.0×10^7^ cells in the BA as described. Scaffolds were maintained for seven days in a tissue culture incubator. During this time, they were continuously perfused with complete MCDB-131 via the aorta, and the flow rate was progressively increased from 1 to 3 mL/min over three days. For a subset of studies, we examined cell viability of RAECs delivered by the IVC route alone; we re-endothelialized scaffolds by stopping aortic perfusion, cannulating the IVC, and then infusing 3.0×10^7^ RAECs. In these studies, after IVC cell delivery, scaffolds were returned to a tissue culture incubator and maintained under retrograde aortic perfusion without receiving any additional cells through the aorta or BA.

### Histology and cell nuclei/vessel quantification

The re-endothelialized scaffolds were dissected into four short axis views that were evenly spaced between the base and the apex of the heart. The dissected scaffolds were then paraffin embedded and sectioned (5 µm). After being rehydrated, sections were stained with hematoxylin and eosin (H&E) or Verhoeff-Van Gieson stain. To determine cellularity, 4′,6-diamidino-2-phenylindole (DAPI; Vectorlabs)-stained nuclei were quantified and normalized to the tissue area. To quantify vessel diameter and elastin positivity, Verhoeff-Van Gieson-stained scaffold sections were analyzed. The diameter of re-endothelialized vessels was obtained by measuring the short axial diameter of cell-containing vessels with ImageJ software (NIH), and the number of elastin-positive versus elastin-negative vessels was recorded for each delivery strategy. We used DAPI staining of serial paraffin-embedded sections to confirm that cell nuclei were relining the vessels. All imaging was performed using a Nikon Eclipse TE200 inverted microscope (Fryer Co. Inc.). In the nuclei quantification, vessel diameter, and elastin positivity studies, images were evenly distributed between the different short axis cross-sectional views of the re-endothelialized scaffolds to assess cell distribution across the whole scaffold, and a total of 20 images were analyzed.

### Cell labeling for tracking and viability studies

Cell tracking was performed by using a montage of fluorescent images of labeled cells. Briefly, RAECs were labeled with the lipophilic tracers DiI or DiO on the day of re-endothelialization. The medium (complete MCDB-131) was removed from a confluent plate of RAECs and replaced with Dulbecco's PBS containing 5 µM SP-DiIC18 or SP-DiOC18 (Life Technologies). The plates were incubated for five minutes at 37°C and then for 15 minutes at 4°C. We washed the plates once with PBS and then added culture medium; the cells were incubated for two hours at 37°C and then trypsinized and used to re-endothelialize the scaffolds. After one week of *in vitro* growth, the re-endothelialized scaffolds were removed from the incubator and imaged on a Stereo Discovery V20 Macro Stereo (Carl Zeiss Inc.). Then, they were dissected, placed in Slowfade (Life Technologies), and photographed on a 510 Meta Confocal microscope (Carl Zeiss Inc.).

To validate cell viability, RAEC-seeded scaffolds were labeled with the vital dye Cell Tracker Green 5-chloromethylfluorescein diacetate (CMFDA; Life Technologies) on the last day of culture (day 7). We removed the complete culture medium, added serum-free CMFDA-containing DMEM (Cellgro), and circulated the medium for 45 minutes at 37°C. Then, we replaced the CMFDA-containing medium with complete MCDB-131and circulated the medium for an additional 45 minutes at 37°C. The scaffolds containing CMFDA-labeled cells were removed from the incubator, dissected, and placed in Slowfade (Invitrogen); live cells that had converted CMFDA to a fluorescent agent were imaged on a 510 Meta Confocal microscope.

### Glucose-6-phosphate dehydrogenase activity assay

Cell death was monitored by quantifying the release of glucose-6-phosphate dehydrogenase (G6PDH) into the medium by damaged and dying cells. Medium (1 mL) was harvested daily from the perfusate of the cultured scaffolds and stored at −20°C. On the day of the assay, samples were thawed, and G6PDH activity was quantified using the Vybrant Cytotoxicity Assay Kit (Life Technologies), according to the manufacturer's instructions.

### Terminal deoxynucleotidyl transferase dUTP nick end labeling (TUNEL) assay

The DeadEnd Colorimetric TUNEL system (Promega) was used to stain for nicked DNA in paraffin-embedded sections of re-endothelialized hearts to detect dying cells. We modified the manufacturer's instructions as follows: after the samples were deparaffinized and rehydrated, they were microwaved for two minutes in a 10 mM citrate buffer solution [25], and the samples were incubated with DyLight 594-conjugated streptavidin (Jackson ImmunoResearch). The slides were mounted with Vectashield mounting medium containing DAPI and imaged using a Nikon Eclipse TE200 inverted microscope (Fryer Co. Inc.).

### In vitro thrombomodulin assay

To assess scaffold thrombogenicity or the potential of the scaffolds to clot, we examined protein C activation as a surrogate for activation of the anticoagulation pathway. We adapted a previously described endothelial cell thrombomodulin assay for our studies [26], [27]. Briefly, on the last day of culture, scaffolds were washed three times by retrograde perfusion of phenol red-free DMEM/F12 (Life Technologies) at 1 mL/min for a total of 45 minutes (15 minutes per wash) through the aorta. Then, we continuously circulated 4 mL of phenol-red free DMEM/F12 containing human α-thrombin (0.1 NIH U/mL, Haematologic Technologies) and human protein C (12 µg/mL, Haematologic Technologies) retrograde through the aorta of the scaffolds for 45 min at 1 mL/min. We transferred 100 µL of the medium in triplicate to a 96-well plate; sample-containing wells were mixed with 50 µL of hirudin stock (12 ATU/mL, American Diagnostica), and then the plate was incubated for five minutes at 37°C. Next, the substrate S-2366 (Chromogenix) was added to a final concentration of 0.75 mM, and the plate was incubated at room temperature for five minutes. Finally, the absorbance at 410 nm and 490 nm was measured using a Spectra MAX 340 (Molecular Devices). The relative absorbance was calculated (A_490_-A_410_) and normalized to the relative absorbance measured for acellular scaffolds

### Immunofluorescence staining

Paraffin-embedded sections from re-endothelialized scaffolds and transplanted scaffolds were rehydrated, and antigen retrieval was performed. Briefly, the slides were boiled in 10 mM citrate buffer with 0.05% Tween-20 at pH 6.0 for 20 minutes and then blocked in 3% BSA in PBS for one hour. Then, we incubated the slides with 10 ug/mL of the appropriate primary antibody in PBS overnight at 4°C. We used antibodies to proliferating cell nuclear antigen (PCNA) (rabbit polyclonal, Santa Cruz), CD31 (rabbit polyclonal, Santa Cruz), endothelial nitric oxide synthase (eNOS), calretinin, vimentin (rabbit polyclonal, Abcam), vascular endothelial growth factor receptor 2 (VEGFR2; mouse monoclonal, BD Bioscience), CD34, CD45 (mouse monoclonal, Santa Cruz), α-smooth muscle actin (mouse monoclonal, Sigma), and von Willebrand factor (vWF; rabbit polyclonal, Abcam; goat polyclonal, Santa Cruz). The slides were washed between steps with three changes of PBS containing 0.05% Tween-20 and incubated for one hour with the appropriate secondary antibody conjugated with either FITC or Texas Red (Jackson Immunoresearch) at a 1∶250 dilution. We mounted the slides with DAPI-containing mounting medium and examined them on a Nikon Eclipse TE200 fluorescent microscope.

### Heterotopic transplantation

After recipient rats were anesthetized, we made a midline incision in the abdominal wall to expose the descending aorta and IVC. We performed an end-to-side anastomosis of the donor heart's ascending aorta and left pulmonary artery to the recipient rat's abdominal aorta and vena cava with 9-0 suture as described [28]. Recipient rats were heparinized before transplantation and received continued anti-coagulation therapy (sodium heparin, 100 IU/Kg twice on day of transplant, 200 IU/Kg subcutaneous for the next two days) and daily Coumadin (0.25 mg/Kg) in the drinking water. One week after transplantation, transplanted scaffolds were recovered, dissected into 4 short-axis sections, and paraffin embedded for histologic analysis.

### Isolation of rat neonatal cardiac cells

We isolated rat neonatal cardiac cells following previously published methods [20]. Briefly, Fischer-344 rats (1 to 3 days old) were anesthetized with 5% isoflurane (Abbott Laboratories). We excised the hearts under sterile conditions and placed them into 50-ml conical tubes (on ice). The hearts were dissociated, and cardiac cells were isolated using a Neonatal Cardiomyocyte Isolation System kit (Worthington Biochemical) according to the manufacturer's guidelines. The neonatal cardiac cells were suspended in a small volume of medium (approximately 1 mL) consisting of Iscove's Modified Dulbecco's Medium (Life Technologies) with 10% FBS (HyClone), 2% horse serum (Life Technologies), 100 U/ml penicillin (Life Technologies), 100 U/ml streptomycin (Life Technologies), 2 mmol/l L-glutamine (Life Technologies), 0.1 mmol/l 2-mercaptoethanol (Life Technologies), 1.2 mM CaCl (Fisher Scientific), and 0.8 mM MgCl (Sigma).

### Left ventricle construct recellularization and functional evaluation

Hearts were decellularized, and 4×10^7^ RAECs were infused into the BA. To allow for cell attachment and proliferation, constructs were maintained for seven days in a tissue culture incubator as described above. Next, the left atrium was cannulated, and constructs were mounted in a working heart bioreactor based on the water-jacketed working heart system (Radnoti Glass). We perfused the constructs with neonatal cardiomyocyte culture medium at an atrial flow rate of 20 mL/min. Under retrograde perfusion, rat neonatal cardiac cells (1.3×10^8^ cells) were injected into the left ventricular wall in three to four parallel injections, and the recellularized constructs were maintained by using retrograde Lagendorf perfusion, as previously described [20]. One day after injections, we sutured sterile electrodes to the apex and base of the constructs, which were paced continuously at a frequency of 1 Hz with 6 ms 45–60 V pulses and a delay of 170 ms using a Grass SD-9 stimulator (Grass Medical Instruments). The constructs were maintained for a total of 10 days after left ventricular recellularization (9 days with pacing); on the last day of culture, a microtip pressure catheter was inserted into the left ventricle to assess construct function. Pressure generation was monitored as we gradually increased the pacing frequency from 1 Hz to 4 Hz. Measurements were recorded by using a Powerlab 16-channel data acquisition system (AD Instruments) and Chart 5.3 (AD Instruments). After functional assessment, the constructs were dissected, and the sections were stained with H&E as described. Control constructs did not receive RAECs before injection of rat cardiac-derived cells into the left ventricular wall.

## Results

### Optimizing cell perfusion strategy improves re-endothelialization of decellularized whole hearts

**Figure 1:**
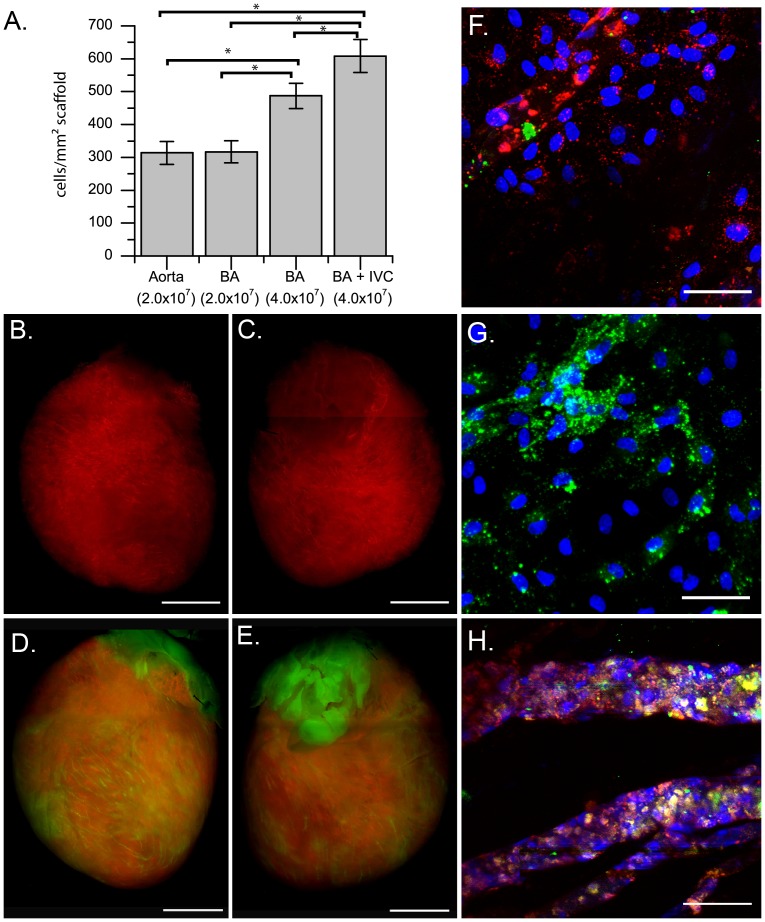
Cellularity and localization of labeled RAECs. (A) Number of DAPI-positive nuclei per mm^2^ of scaffold for each delivery method: the aorta only (Aorta), the BA only (BA), or the combined IVC+BA method (n = 3 hearts per method; mean ±SEM). The total number of cells delivered is indicated in parenthesis. (B, C) Image of whole heart in which 4×10^7^ DiI-labeled RAECs were delivered via the BA. (D, E) Image of whole heart in which 2×10^7^ DiO-labeled cells were delivered via the IVC followed by an additional 2×10^7^ DiI-labeled cells administered via the BA. View of the (F) left ventricular endocardial surface, (G) right ventricular endocardial surface, and (H) the ventricle wall of a heart scaffold recellularized via the BA+IVC cell delivery technique with cells labeled as in D and E. DAPI-positive nuclei are blue (F–H), and overlapping green and red staining shows as yellow (D–H). *p<0.05. Scale bars represent 5 mm (B–E) and 50 microns (F).

Cells may be delivered through the vasculature via two routes: arterial or venous. To determine the optimal strategy for re-endothelialization, we examined three different cell delivery methods: arterial infusion through the aorta, arterial infusion through the BA, or a combined venous and arterial infusion of cells through the IVC and BA (Figure 1). At one week after cell delivery, cells were dispersed throughout the scaffolds regardless of delivery route. We found no statistically significant difference in the number of endothelial cells in the matrix when 2×10^7^ cells were delivered via the aorta or the BA (Figure 1A). The number of cells seen in the heart after seven days increased significantly when 4×10^7^cells were delivered (Figure 1A); this finding indicates that RAEC attachment and growth was not limited by the available luminal space of the acellular scaffolds when 2×10^7^cells were delivered. The greatest cellularity was observed when the combined venous and arterial delivery route was used. Scaffolds seeded with cells delivered using the IVC+BA route had significantly more cells than did scaffolds re-endothelialized with the same number of cells delivered via arterial infusion (Figure 1A), indicating that the IVC+BA cell delivery method enabled greater cell growth. Although no statistical difference was seen in the total number of cells present between the aortic or BA delivery methods, histologic study showed that cells delivered via the aorta only were not uniformly distributed throughout the heart (data not shown). Thus, we used the arterial BA delivery method and the combination IVC+BA cell delivery methods as our main re-endothelialization strategies for the remaining experiments.

Labeling RAECs with DiI and DiO before recellularization further confirmed the uniform distribution of cells throughout the heart matrix after BA (Figure 1B, C) or combined IVC+BA delivery (Figure 1D, E). We examined the distribution of cells delivered by the IVC+BA re-endothelialization approach in greater detail to determine if cells delivered by either the venous or arterial route preferentially recellularized different regions of the scaffold (Figure 1F, H). The endocardial surface of the left ventricle was predominantly recellularized with RAECs delivered via the BA (Figure 1F), whereas the endocardial surface of the right ventricle was populated with RAECs delivered via the IVC (Figure 1G). Furthermore, vessels predominantly re-endothelialized by cells from a single route were observed (Figure S1), and cells delivered from both routes were seen to co-localize in some vessels (Figure 1H) in the ventricular free walls. This finding suggests that some vascular conduits are connected.

**Figure 2:**
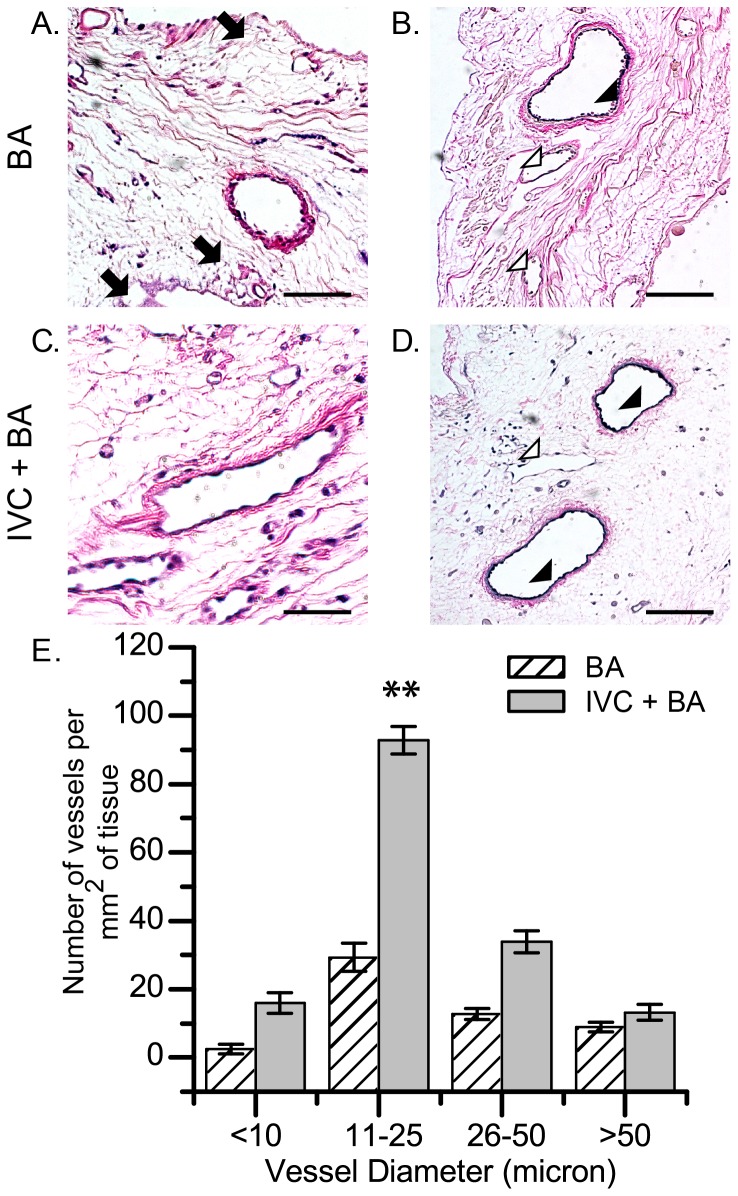
Histologic assessment of decellularized rat heart scaffolds seeded with RAECs. (A,C) H&E and (B,D) Verhoeff-Van Gieson staining of scaffolds recellularized by the BA only technique (A,B) and the combined IVC+BA technique (C,D); arrows indicate cell-free vessels (A), arrow heads indicate elastin-positive vessels with cell nuclei (B,D), and open arrow heads indicate elastin-negative vessels (B,D). All scaffolds were recellularized with 4×10^7^ RAECs. (E) Quantification of the number of vessels lined by DAPI-positive cell nuclei in the mid-ventricular wall; the results are grouped according to vessel diameter (n = 3 per data set; mean ±SEM). **p<0.001 for IVC+BA vs BA re-endothelialization techniques for vessels with a diameter of 11–25 microns. Scale bars represent 125 microns (A–D).

Scaffolds seeded with cells delivered using arterial cell perfusion via the BA or combined venous and arterial cell perfusion via the IVC and BA were examined to determine if there was a correlation between cell delivery technique and the types of vessels that were re-lined (i.e., size and elastin positivity). H&E and Verhoeff-Van Gieson staining of re-endothelialized scaffolds showed that vessels of varying diameters were re-lined with RAECs (Figure 2A, C) as were both elastin-positive arterial vessels and elastin-negative vessels (Figure 2B, D). We found no statistically significant differences in preference for either elastin-positive or elastin-negative vessels between arterial only (BA) and venous and arterial (IVC+BA) cell delivery (data not shown). Regardless of delivery technique, RAECs maintained a flattened morphology and did not occlude vessel lumens (Figures 2A–D). Quantification of vessel diameter within the ventricular wall showed that the combined venous and arterial (IVC+BA) delivery of cells resulted in a statistically significant increase in re-lined small vessels (11 to 25 microns in diameter) in the mid-ventricular wall compared to arterial (BA) delivery alone (Figure 2E). This difference in vessel diameter distribution was not seen in apical sections (data not shown).

### Rat aortic endothelial cells survive in re-endothelialized scaffolds

**Figure 3:**
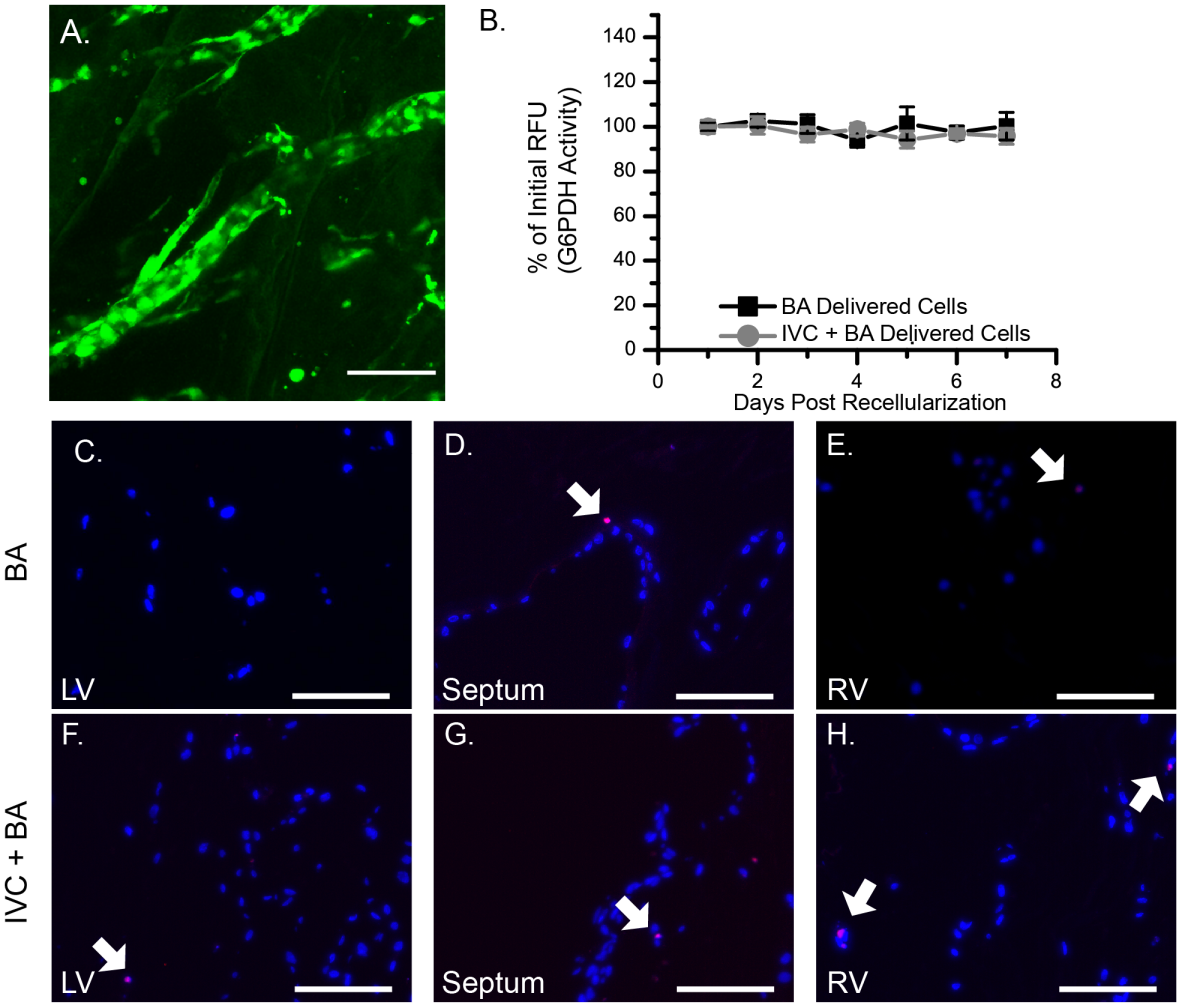
Cell survival in re-endothelialized heart scaffolds. (A) CMFDA-labeled cells (green) in the ventricle wall of a scaffold recellularized with 4×10^7^ RAECs via the BA technique and cultured for seven days before CMFDA labeling.(B) Quantification of G6PDH activity in the medium as an indicator of cell viability over time expressed as a percent of the initial relative fluorescence unit (RFU) measured using the Vybrant Cytotoxicity Assay Kit (n = 6 for each re-endothelialization technique; results are expressed as mean ±SEM).(C–H) TUNEL staining of scaffolds re-endothelialized with 4×10^7^ RAECs after seven days of culture. Images of the (C) left ventricle (LV), (D) septum, and (E) right ventricle (RV) of scaffolds seeded using the BA cell delivery technique. Images of the (F) left ventricle, (G) septum, and (H) right ventricle of scaffolds seeded using the IVC+BA cell delivery technique. (C–H) Cell nuclei are stained with DAPI (blue), and TUNEL-positive staining is red (arrows). Scale bars represent 100 microns.

Acellular scaffolds generated by detergent perfusion create a construct that is chemically complex and structurally thick. Endothelial cells can potentially be delivered to regions within the scaffold that are not efficiently fed by the medium using retrograde aortic perfusion and must rely on diffusion of nutrients. This scenario can lead to cell death. To assess RAEC survival and quantify cell death in recellularized scaffolds, we used three different assays: (1) CMFDA cell labeling at the end of seven days of *in vitro* culture, (2) quantification of G6PDH activity released in the scaffold perfusate over a seven-day period, and (3) TUNEL staining at the end of seven days of *in vitro* culture. CMFDA is a non-fluorescent molecule that is cleaved by metabolically active, viable cells to produce a green-fluorescing product. We found fluorescent CMFDA-labeled cells lining both large and small vessels in the ventricle wall (Figure 3A), indicating that the RAECs that re-lined the vascular conduits were viable after a week of *in vitro* culture. CMFDA-positive RAECs also lined the endocardial wall and trabeculae (Figure S2). Cell viability measured by CMFDA was not dependent on the delivery route, and retrograde aortic media perfusion was sufficient to maintain cell viability because scaffolds re-endothelialized via the IVC-only route still had CMFDA-positive cells after one week of culture (Figure S2). We also quantified G6PDH activity as an indicator of ongoing cell death. No statistically significant increases were observed in G6PDH activity during seven days of *in vitro* culture; G6PDH activity at one day after cell seeding was 100±0.3% compared with 95.9±1.03% at seven days post-seeding, regardless of whether the cells were delivered using the BA only or the IVC+BA route (Figure 3B). We analyzed the sensitivity of the assay and found that the lower limit of detection of G6PDH was 12–30 cells/mL. Finally, TUNEL analysis demonstrated no significant apoptosis of RAECs on day seven (Figure 3C–H) regardless of cell location (left or right ventricle or septum) or delivery method (BA or IVC+BA). Together, these results indicate that aortic perfusion is sufficient to maintain RAECs throughout a re-endothelialized scaffold for seven days after cell delivery, regardless of delivery method and location within the scaffold.

### Rat aortic endothelial cells proliferate and maintain anti-coagulant properties in re-endothelialized scaffolds

**Figure 4:**
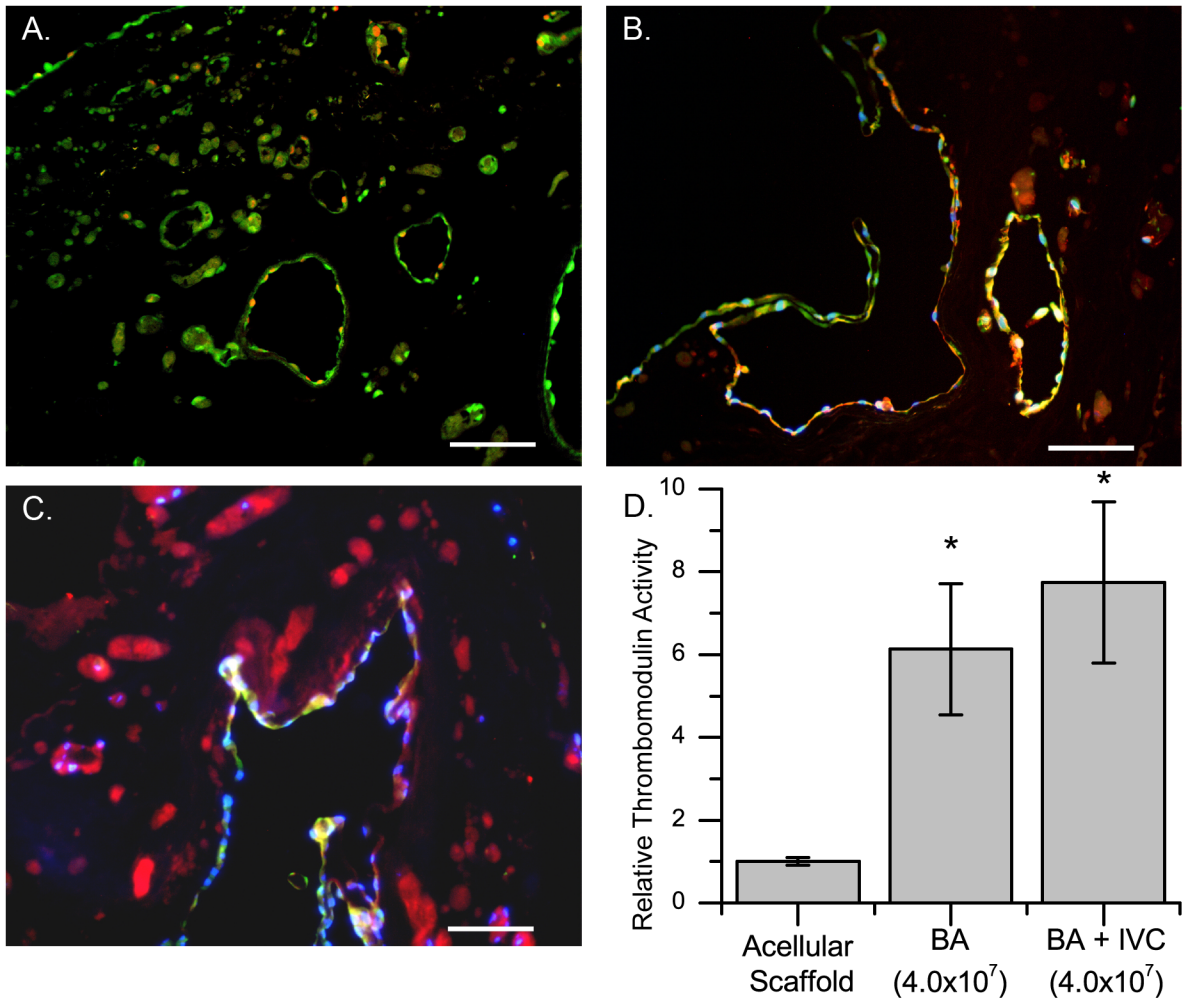
Functional analysis of re-endothelialized scaffolds after seven days of cell culture. Scaffolds recellularized via the BA showing (A) CMFDA (green) and PCNA (red) staining; (B) CMFDA (green), eNOS (red), and DAPI (blue) staining; and (C) CMFDA (green), vWF (red), and DAPI (blue) staining; yellow staining indicates the combination of green and red. (D) Thrombogenicity of re-endothelialized matrices expressed as a ratio of recellularized to acellular controls (n = 6 for acellular controls, n = 8 for BA and BA+IVC re-endothelialized scaffolds). The total number of RAECs delivered is indicated in parentheses. *p<0.05, re-endothelialized scaffolds vs acellular controls; results are expressed as the mean ±SEM. Scale bars represent 100 microns (A–C).

It is important that the cells in re-endothelialized scaffolds are uniformly distributed and remain not only viable but also functional. RAEC phenotype and function were examined by immunofluorescence staining of re-endothelialized scaffolds seven days after RAEC delivery. PCNA^+^ cells were seen throughout the scaffold, suggesting that cells retained their ability to proliferate (Figure 4A). Likewise, eNOS^+^ cells were found throughout the vascular tree, indicating that the cells remained functional (Figure 4B). Lastly, RAECs expressed vWF (Figure 4C), indicating the potential for regulating coagulation. To determine if re-endothelialized scaffolds could inhibit the coagulation pathway, we performed an *in vitro* thrombomodulin assay on scaffolds seven days after re-endothelialization. We observed a 6 to 8-fold statistically significant increase in thrombomodulin and thrombin-mediated protein C activity in re-endothelialized scaffolds compared with acellular ones (Figure 4D). This finding suggests that recellularized scaffolds can inhibit the coagulation cascade because they retain the capacity to activate protein C, which negatively regulates the coagulation cascade [26], [27]. Thrombomodulin assay results were similar for scaffolds that had been recellularized via the BA or IVC+BA cell delivery methods (Figure 4D).

### Re-endothelialization of heart scaffolds before heterotopic transplantation reduces clotting

**Figure 5:**
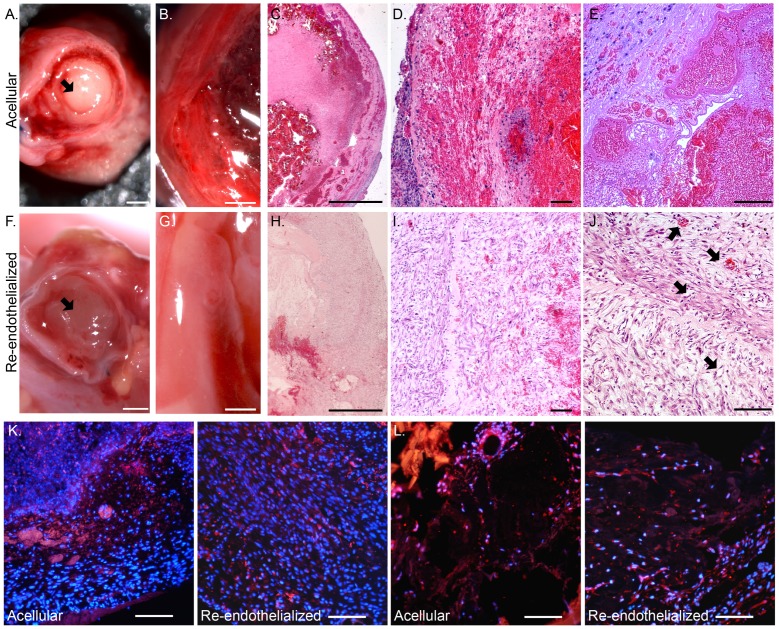
Characterization of heterotopic transplants. (A–E) Acellular and (F–J) re-endothelialized scaffolds seven days after heterotopic transplantation. Short axial view of an aorta with blood clot (arrow) from an acellular scaffold (A) and a non-clotted aorta (arrow) from a re-endothelialized scaffold (F) after transplantation. Short axial view of the ventricle wall of an acellular scaffold with a blood clot (B) and a re-endothelialized scaffold (G). H&E staining of a transplanted acellular scaffold (C–E) with a blood clot inside the ventricular cavity and a re-endothelialized scaffold (H–J) at increasing magnification (2X, 10X, and 20X). Arrows point to patent vessels with and without blood (J). CD31 (K) and VEGFR2 (L) (red) staining in transplanted scaffolds; DAPI-positive nuclei are blue. Scale bars represent 1 mm (A–C and F–H) and 100 microns (D, E, I, J, K, and L).

To determine whether RAECs in re-endothelialized scaffolds remained functional and could reduce clotting *in vivo*, we heterotopically transplanted acellular scaffolds or those that had been re-endothelialized with RAECs using the BA cell delivery method into the abdomen of recipient rats. The re-endothelialized scaffolds were cultured *in vitro* for seven days before transplantation. Then, seven days after *in vivo* transplantation, we explanted the scaffolds for examination (Figure 5).We found less aortic clotting in re-endothelialized scaffolds (Figure 5F) than in acellular scaffolds (Figure 5A). Examination of the left ventricular wall and ventricular cavity showed greater thrombogenesis in the acellular scaffold transplants than in the re-endothelialized scaffolds (Figure 5B and 5G). A wider tissue distribution of blood cells was observed in the parenchyma of the acellular scaffolds than in re-endothelialized scaffolds (Figure 5B–E and 5G–J) as shown by the intense red H&E staining coloration. Patent vessels with and without blood were observed only in re-endothelialized scaffolds (Figure 5J).

We used immunofluorescence staining to characterize the cells present in the transplanted scaffolds. The majority of the cells stained positive for CD31 and VEGFR2 suggesting the presence of endothelial cells in the heterotopic transplant, even for acellular scaffolds that were not seeded with RAECs before transplantation (Figure 5K, L). The progenitor cell markers CD34 and CD45 were expressed only by a small subset of the recruited cells in both the acellular and re-endothelialized scaffolds. Heterotopic transplantation of either acellular or re-endothelialized heart scaffolds did not lead to significant staining of smooth muscle actin, vimentin, or calretinin, which are common markers for smooth muscle cells, fibroblasts, and mesothelium, respectively (data not shown).

### Re-endothelialization before recellularization of the left ventricle wall improves contractility of the heart construct

**Figure 6:**
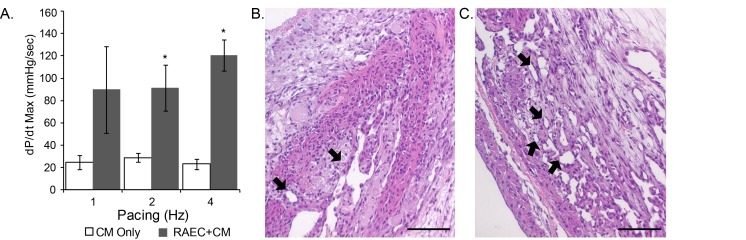
Effect re-endothelialization before left ventricle wall recellularization on contractility of the heart construct. (A) Maximal rate of change in pressure (dP/dt max) of the left ventricle at different pacing frequencies in constructs that were re-endothelialized with rat aortic endothelial cells and recellularized with rat neonatal cardiac cells (RAEC+CM) and control constructs in which neonatal cardiac cells were injected into the left ventricle wall without prior re-endothelialization (CM only). (B–C) H&E staining of rat neonatal cardiac cells in the left ventricle of a scaffold without (B) and with re-endothelialization (C). Arrows indicate re-lined vessels in the recellularized left ventricle. Scale bars represent 100 micron; n = 3 in each group; *p<0.05, vs control constructs; the results are expressed as the mean ±SEM.

To further characterize the functional benefits of using re-endothelialized scaffolds, we examined the effects of re-endothelialization on the function of beating heart constructs in which the left ventricle was recellularized with neonatal cardiac cells. At a pacing frequency of 2 to 4 Hz, the average maximal rate of change in pressure was significantly higher in re-endothelialized constructs than in constructs that had not been re-endothelialized before left ventricle-recellularization (Figure 6A). H&E staining revealed that constructs that were first re-endothelialized had more re-lined vessels within the recellularized myocardium than scaffolds that were not re-endothelialized (Figure 6B–C). In the few relined vessels seen in constructs that were not re-endothelialized with RAECs before left ventricle-recellularization, the cells lining the vessels were likely endothelial cells present in the cardiac cell isolations, which is consistent with our previous finding (Figure 6B) [20].

## Discussion

In this study, we re-endothelialized the vascular network of whole rat heart acellular scaffolds with RAECs delivered via aortic, BA only, or combination IVC and BA infusion. Regardless of the method used to deliver the cells into decellularized heart scaffolds, RAECs lined the coronary vessel conduits and proliferated along the vessel conduit walls without penetrating into the parenchyma of the scaffolds. The combined use of arterial and venous cell delivery resulted in a superior distribution of re-lined vessels in the mid-ventricular free wall. Moreover, for both arterial and venous cell delivery, retrograde aortic perfusion of cell culture medium through the recellularized coronary arterial vessels supported RAECs that were seeded onto the venous side of the coronaries with no significant cell apoptosis (Figure 3). Finally, re-endothelialized scaffolds had anti-coagulant properties and decreased *in vivo* thrombogenicity (Figure 4 and 5), and re-endothelialization improved the performance of scaffolds that were recellularized with neonatal cardiac cells (Figure 6).

In this study, we found that cells delivered by arterial routes were more homogeneously distributed throughout the scaffold (from the base to the apex) when they were infused into an established retrograde aortic flow of medium. The need to infuse cells into a scaffold under retrograde perfusion may relate to a greater vessel resistance in arterial vessels. The vessels on the arterial side of the heart are subjected to greater shear stress than are vessels on the venous side and thus have elastin in the basement membrane, which could also increase their resistance to cell infusion. On the venous side of the heart, shear stress is less, and the vessels on this side may be more easily infused with cells.

Determining the degree to which the capillary beds have reformed is challenging in structurally complex scaffolds. However, using labeled cells, we found that cells delivered via the IVC and the BA localized to the same vessels in some instances, suggesting the presence of a continuous conduit between the arterial and venous coronary vessels in these scaffolds (Figure 1H). This venous-arterial interconnectivity is probably not due to the presence of capillary beds because the decellularization process should remove these one-cell thick vessels. Instead, co-localization of the endothelial cells may occur in metarterioles, or alternatively, the endothelial cells delivered to the venous and arterial sides of the acellular coronary vessels may anastomose to re-form nascent capillary beds. This generation of small patent tubules is well documented *in vitro* when endothelial cells are cultured on a supportive matrix [29].

Because these scaffolds are derived from cadaveric hearts under conditions that preserve the architecture and biological cues associated with the native matrix, they retain glycosaminoglycans (GAGs), growth factors, and structural cues, all of which could be advantageous for recellularization. For example, retained GAGs may bind pro-angiogenic heparin-binding growth factors that increase cell recruitment [27] and could account for the large number of CD31^+^ and VEGFR2^+^ cells observed in the scaffolds (Figure 5K and 5L). Identifying the factors that contribute to cell recruitment and differentiation will be critical to developing an intact functional, vascularized organ.

Although GAGs and some growth factors are likely present on the scaffold, supplementing the matrix with other GAG-associated proteins may improve cell recruitment, proliferation, differentiation, and viability. Stromal cell-derived factor 1 (SDF-1) and VEGF are good candidates for this approach because they promote cell recruitment and angiogenesis, respectively, and are known to associate with GAGs [30], [31]. In fact, our studies may have benefited from proteins that absorbed into the matrix from the serum in the medium before re-endothelialization and during the subsequent culture period.

Re-endothelialization of cardiac tissue with RAECs before delivering neonatal cardiac cells to the left ventricle improved cardiac contractility *in vitro* (Figure 6). The RAECs may have created better functioning constructs by enhancing cardiomyocyte organization and survival. Endothelial cells, when co-cultured *in vitro* with cardiomyocytes in 2D cultures, have been shown to promote increased cardiomyocyte organization and survival [32]. In addition, re-endothelialization of the construct before recellularization of the left ventricle may have improved contractility by enhancing nutrient transport to the neonatal cardiomyocytes, which in turn may have also enhanced cell maturation. These results indicate that identifying the proper combination of cell types (vasculogenic versus myogenic) and the order of delivery will be critical to the optimal performance of recellularized whole organs. Moreover, our findings suggest that for the current model re-endothelialization before left ventricle recellularization is optimal.

In summary, we have shown that re-endothelialization of a whole heart scaffold is optimal when cells are delivered through both venous and arterial coronary vessels. Moreover, we found that re-endothelialization reduces scaffold thrombogenicity and enhances the function of a left ventricle–recellularized construct. Our findings lay the groundwork for generating whole-heart scaffolds or cardiac patches that have perfusable, non-thrombogenic vessels. Future work will involve the use of additional cells for recellularization of constructs in order to create a fully functioning vasculature that responds to hemodynamic changes.

## Supporting Information

Figure S1 **Localization of labeled RAECs in re-endothelialized scaffolds.** Scaffolds were perfused with 2×10^7^ DiO-labeled RAECs (green) via the IVC, followed by perfusion of 2×10^7^ DiI-labeled RAECs (red) via the BA, and were cultured for seven days. Vessels predominantly lined with RAECs cells delivered via the BA (A) or the IVC (B). DAPI- positive nuclei are blue. Scale bar represents 50 microns.
(TIF)

Figure S2 **CMFDA labeling of RAECs in scaffolds re-endothelialized via the IVC only.** Scaffolds seeded with 3×10^7^ RAECs were labeled with CMFDA on the last day of culture (day 7). CMFDA-positive cells in the ventricle wall (A) and on the endocardial surface (B). Scale bar represents 100 microns.
(TIF)
