# Supplementary figures and images for: Optimizing Recellularization of Whole Decellularized Heart Extracellular Matrix
Source: PLoS One. 2014 Feb 27;9(2):e90406. doi: 10.1371/journal.pone.0090406 (PMC3937369; doi:10.1371/journal.pone.0090406)

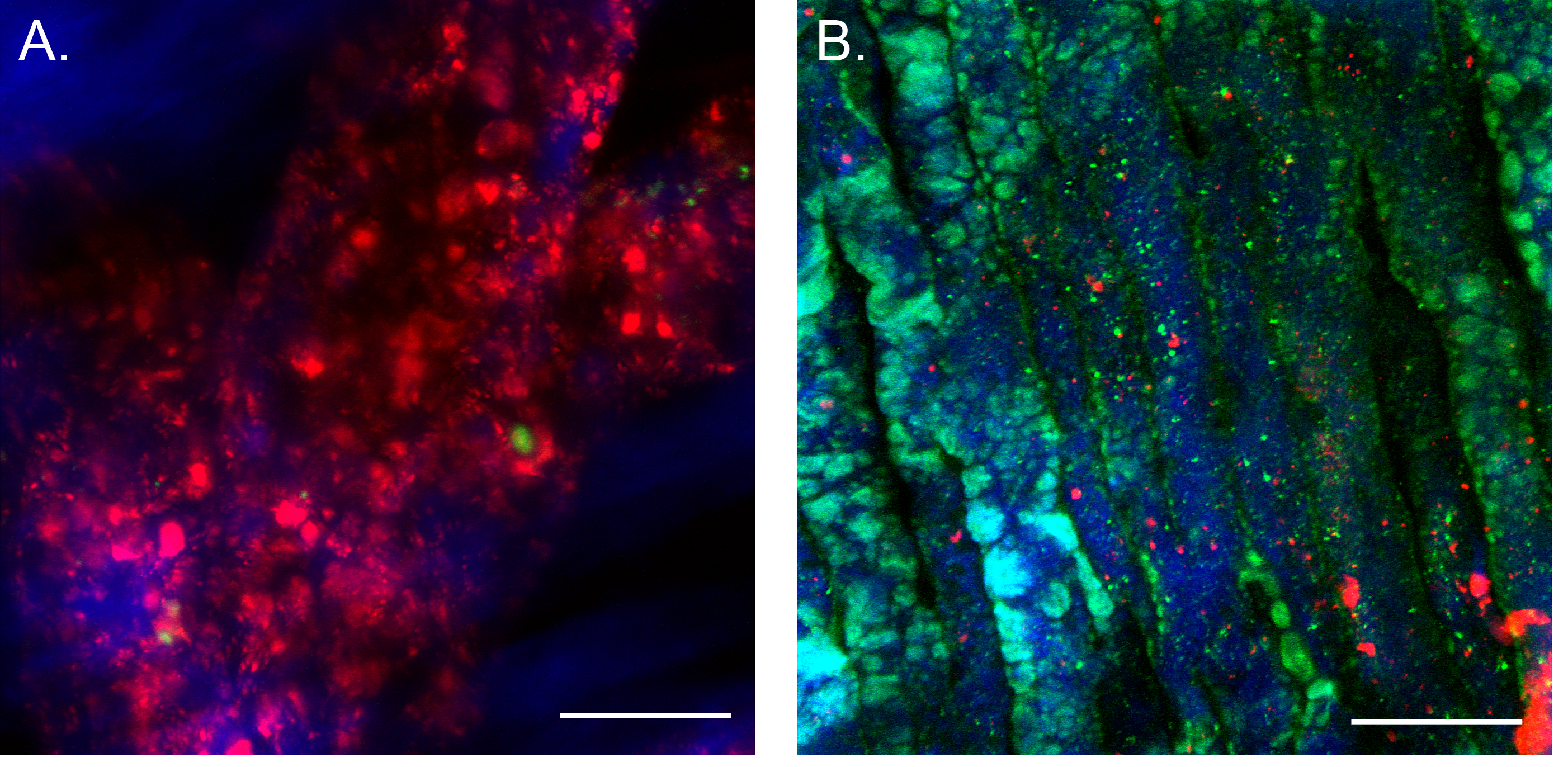

Supplement: Figure S1 — Localization of labeled RAECs in re-endothelialized scaffolds. Scaffolds were perfused with 2×10^7^ DiO-labeled RAECs (green) via the IVC, followed by perfusion of 2×10^7^ DiI-labeled RAECs (red) via the BA, and were cultured for seven days. Vessels predominantly lined with RAECs cells delivered via the BA (A) or the IVC (B). DAPI- positive nuclei are blue. Scale bar represents 50 microns. (TIF) [file pone.0090406.s001.tif]

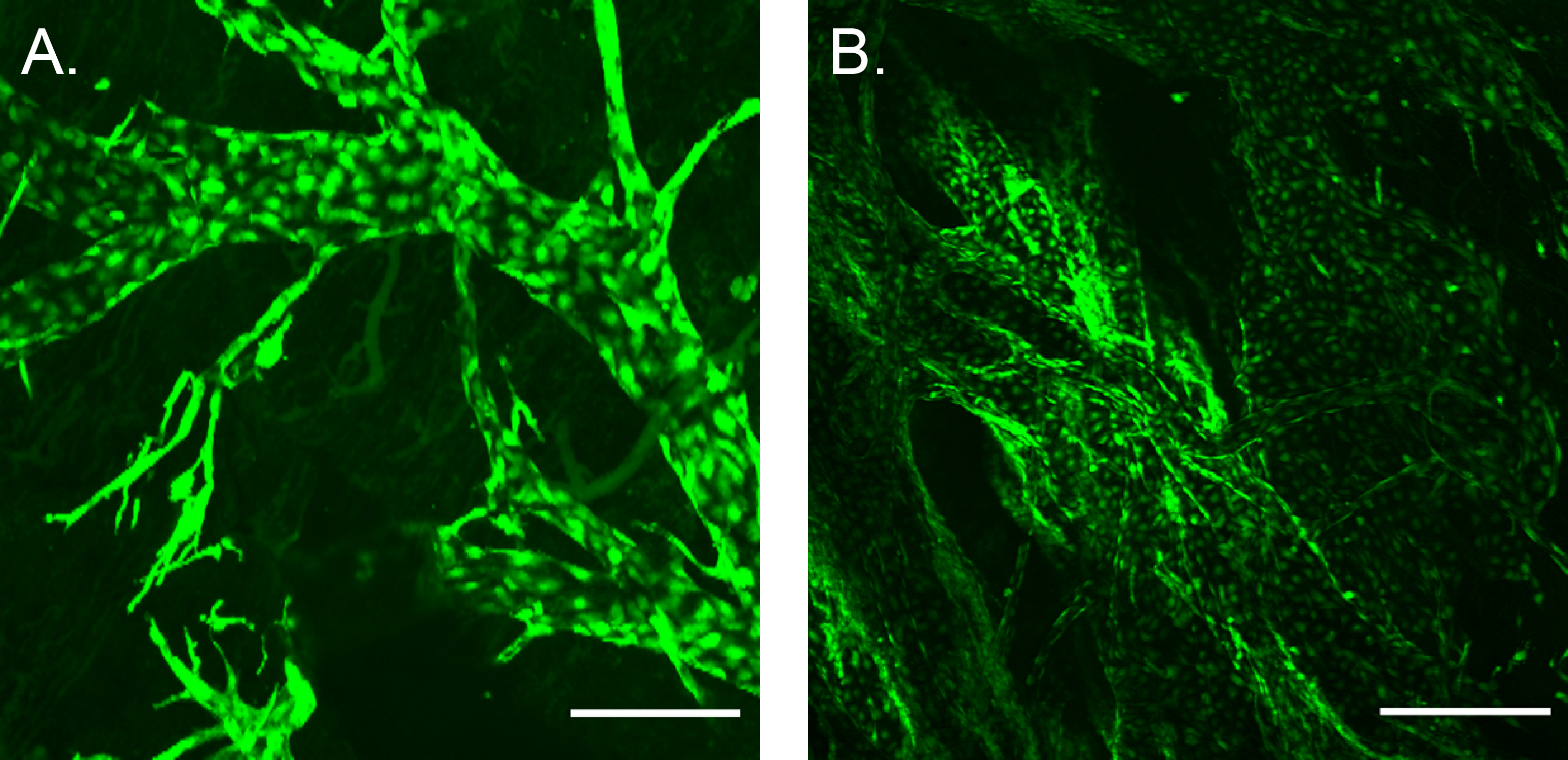

Supplement: Figure S2 — CMFDA labeling of RAECs in scaffolds re-endothelialized via the IVC only. Scaffolds seeded with 3×10^7^ RAECs were labeled with CMFDA on the last day of culture (day 7). CMFDA-positive cells in the ventricle wall (A) and on the endocardial surface (B). Scale bar represents 100 microns. (TIF) [file pone.0090406.s002.tif]
